# Influence of salinity on the diversity and composition of carbohydrate metabolism, nitrogen and sulfur cycling genes in lake surface sediments

**DOI:** 10.3389/fmicb.2022.1019010

**Published:** 2022-11-28

**Authors:** Qing Liu, Jian Yang, Beichen Wang, Wen Liu, Zhengshuang Hua, Hongchen Jiang

**Affiliations:** ^1^State Key Laboratory of Biogeology and Environmental Geology, China University of Geosciences, Wuhan, China; ^2^National and Local Joint Engineering Research Center of Ecological Treatment Technology for Urban Water Pollution, Zhejiang Provincial Key Laboratory for Water Environment and Marine Biological Resources Protection, College of Life and Environmental Science, Wenzhou University, Wenzhou, China; ^3^Department of Environmental Science and Engineering, University of Science and Technology of China, Hefei, China; ^4^Qinghai Provincial Key Laboratory of Geology and Environment of Salt Lakes, Qinghai Institute of Salt Lakes, Chinese Academy of Sciences, Xining, China

**Keywords:** biogeochemical cycles, functional gene diversity, functional gene composition, salinity, lake sediments

## Abstract

Exploring functional gene composition is essential for understanding the biogeochemical functions of lakes. However, little is known about the diversity and composition of biogeochemical cycling genes and their influencing factors in saline lakes. In this study, metagenomic analysis was employed to characterize the diversity and composition of microbial functions predicted from genes involved in carbohydrate metabolisms, nitrogen, and sulfur cycles in 17 surface sediments of Qinghai-Tibetan lakes with salinity ranging from 0.7 to 31.5 g L^−1^. The results showed that relative abundances of carbohydrate-active enzyme (CAZy), nitrogen, and sulfur cycling genes were 92.7–116.5, 15.1–18.7, 50.8–63.9 per 1,000 amino acid coding reads, respectively. The Shannon diversity indices of CAZy and sulfur cycling genes decreased with increasing salinity, whereas nitrogen cycling gene diversity showed an opposite trend. Relative abundances of many CAZy (i.e., carbohydrate-binding module and carbohydrate esterase), nitrogen (i.e., anammox and organic degradation and synthesis) and sulfur (i.e., dissimilatory sulfur reduction and oxidation, link between inorganic and organic sulfur transformation, sulfur disproportionation and reduction) cycling gene categories decreased with increasing salinity, whereas some CAZy (i.e., auxiliary activity), nitrogen (i.e., denitrification) and sulfur (i.e., assimilatory sulfate reduction and sulfur oxidation) gene categories showed an increasing trend. The compositions of CAZy, nitrogen, and sulfur cycling genes in the studied lake sediments were significantly (*p* < 0.05) affected by environmental factors such as salinity, total organic carbon, total nitrogen, and total phosphorus, with salinity having the greatest influence. Together, our results suggest that salinity may regulate the biogeochemical functions of carbohydrate metabolisms, nitrogen, and sulfur cycles in lakes through changing the diversity and composition of microbial functional genes. This finding has great implications for understanding the impact of environmental change on microbial functions in lacustrine ecosystems.

## Introduction

Lake sediments are inhabited by a variety of microbial communities, which are widely involved in the biogeochemical cycles of carbon, nitrogen, and sulfur (Haglund et al., 2003; Falkowski et al., 2008; Yang et al., 2020c). It has been demonstrated that the ability of ecological models to predict the biogeochemical processes in ecosystems can be significantly improved by integrating the parameters of microbial functions predicted from genes (Graham et al., 2014; Guo et al., 2020; Albrecht et al., 2021; Chen et al., 2021). Such improvement of the prediction ability of ecological models can be explained by the mechanism that microbial enzymes participate in the regulation of biogeochemical functions in ecosystems (Falkowski et al., 2008). The potential activity of biogeochemical functions of these microbial enzymes can be revealed by their coding genes (Graham et al., 2014; Guo et al., 2020; Chen et al., 2021). Therefore, studying the diversity and composition of functions predicted from genes and their environmental responses in the lake sediments is helpful to understand the biogeochemical functions of lake ecosystems.

The diversity and composition of microbial functions can usually be revealed by cloning library (Yang et al., 2013a), GeoChip (He et al., 2010) or metagenomic analyses (Wang et al., 2022). Metagenomics can provide comprehensive information on the composition of the known and unknown microbial functional genes (Jiang et al., 2018). In recent years, the establishments of some specific functional gene databases have greatly improved the understanding of compositions of functional genes involved in the biogeochemical cycle of carbon, nitrogen, and sulfur. For example, the coding gene database of carbohydrate-active enzymes (CAZy) contains diverse functional enzymes involved in the metabolism and processes of carbohydrates, such as carbohydrate esterase, glycoside hydrolase, glycosyl-transferase, polysaccharide lyase (Lombard et al., 2013); the coding gene databas of nitrogen cycle (NCycDB) includes functional genes involved in nitrogen fixation, nitrification, and denitrification (Tu et al., 2019); the coding gene database of sulfur cycling (SCycDB) consists of functional genes involved in sulfur oxidization, sulfate reduction, and organic sulfur transformation (Yu et al., 2021). Metagenomic analysis based on these specific functional gene databases has been widely employed to characterize diversity and composition of microbial functions predicted from genes involved in carbon, nitrogen, and sulfur cycles in natural environments (Dai et al., 2021; Wang et al., 2022).

Previous metagenomics-based microbial studies mainly focused on surveying microbial amino acid signatures in lakes (Rhodes et al., 2010) or investigating the potential functions of novel microbial species (Ghai et al., 2011; Fernández et al., 2014; Mirete et al., 2016; Vavourakis et al., 2016). Some recent microbial community metagenomic studies have revealed gene profiles related to biogeochemical cycles in freshwater lakes (Xing et al., 2020; Tran et al., 2021). However, similar studies are rare in saline lakes (usually with salinity >1.0 g L^−1^) that are an important part (about half of the total surface area) of the inland aquatic ecosystem (Messager et al., 2016). Saline lakes play important roles in global biogeochemical cycles (Tranvik et al., 2009). Hence, it is necessary to investigate the diversity and composition of microbial functions predicted from genes related to carbon, nitrogen, and sulfur cycles and their responses to environmental changes (e.g., salinity) in saline lakes, and it will avail to comprehensively understand the biogeochemical functions of lacustrine ecosystems.

The taxonomic composition of lake microbial communities is very susceptible to salinity, and varies greatly among lakes with different salinities (Wu et al., 2006; Liu et al., 2016; Yang et al., 2019). Such high variation in the taxonomic composition of microbial communities results in significant different composition of functions predicted from genes (Zimmerman et al., 2013). Thus, it is reasonable to hypothesize that the composition of microbial functions predicted from genes will be very different in lakes with different salinities. However, some studies have also indicated that due to the redundancy of functional genes, large variations in the taxonomic composition of microbial communities may not necessarily be directly reflected as the changes in the composition of microbial functions predicted from genes (Louca et al., 2016, 2018). Therefore, there is still uncertainty about the influence of salinity on the composition of microbial functions predicted from genes involved in the biogeochemical cycles of carbon, nitrogen, and sulfur in lakes.

To fill the abovementioned knowledge gaps, metagenomics was applied to analyze the diversity and composition of microbial functions predicted from genes that are involved in carbohydrate metabolism, nitrogen, and sulfur cycling processes in the surface sediments of seventeen lakes with salinity ranging from 0.7 to 31.5 g L^−1^. The main objectives of this study were to (1) characterize the diversity and compositions of microbial functions predicted from genes related to carbohydrate metabolisms, nitrogen, and sulfur cycles in the studied lake sediments, and (2) quantify the relative importance of environmental factors influencing the composition of microbial functions predicted from genes.

## Materials and methods

### Field sampling

A sampling cruise was carried out in the summer of 2018. In the present study, seventeen sampling sites were chosen from 10 lakes (Supplementary Table S1). The sampling sites are located in the shore zone with water depth of 1–2 meters of each studied lake. Surface sediment (0–5 cm) was collected at each site by using a grab-bucket collection sampler and subsequently was distributed into two 50-ml sterilized tubes using sterile spoons for DNA extraction and geochemical analyses, respectively. The sediment samples were kept on dry ice in the field and during transportation, and then were transferred to a −80°C freezer in the laboratory for further processing.

### Laboratory analysis

The sediment pore water was extracted using centrifugation. The salinity and pH of the sediment pore waters were determined using portable meters (SANXIN, Shanghai, China). The contents of total organic carbon (TOC) and total nitrogen (TN) of the sediments were determined using a multi N/C 2100S analyzer (Analytik Jena, Germany). Freeze-dried sediments were digested with concentrated perchloric acid (12 mol L^−1^) and the resulting extracts were analyzed for the content of total phosphorus (TP) using colorimetric methods (Yang et al., 2020a).

### Metagenomics

Total genomic DNA was extracted from approximately 20 g of wet surface sediments for each sample using the MoBio PowerSoil DNA Isolation kit according to the manufacturer’s instructions. The resulting genomic DNA was sequenced on an Illumina HiSeq 2,500 platform (paired-ends sequencing of 2 × 150 bp) with 350 bp library inserts. Each sample obtained ~30 Gbp of raw paired-end reads. The pair-end raw reads were de-replicated and trimmed using “fastp” with parameters of −D, −q 20 and −l 50 (Chen et al., 2018). The resulting clean reads were assembled individually for each sample in metaWRAP using the “metawrap assembly” function with default settings (Uritskiy et al., 2018). Amino acid coding sequences of all the assembled contigs were predicted using Prodigal (Hyatt et al., 2010) with parameter of *-p meta*. The resulting amino acid translations were annotated using DIAMOND v2.0.11 (Buchfink et al., 2014) with parameters of -blastp, --min-score 60 -k 25 and --id 60 against the CAZy database (Lombard et al., 2013), NCycDB and SCycDB (Tu et al., 2019; Yu et al., 2021). In this study, a total of 25 top hits were generated for each functional gene sequence using the DIAMOND program, and the resulted functional gene with the highest bit scores was manually selected as the reference. Besides, the resulting amino acid sequences were also BLASTed (parameter sets: -blastp, --min-score 60 -k 1 and --id 60) against the NCBI (national center for biotechnology information) NR (non-redundant protein sequence) database to obtain the taxID of each gene. The taxonomy of each functional gene was assigned using *getTaxonomy* function in the R package of “taxonomizr” according to the taxID retrieved from the NR database references (Scott, 2022). The frequency of occurrence of each functional gene was calculated for each sample and then integrated into a table for further statistical analysis. To analyze the diversity of different gene categories (e.g., CAZy, nitrogen and sulfur cycling genes), Shannon index was calculated using *diversity* function in R package of “vegan” (Oksanen et al., 2020). For each sample, the gene counts were normalized to the total number of amino acid coding gene sequences predicted by Prodigal prior to downstream analysis (McKnight et al., 2019; Dove et al., 2022).

### Statistical analysis

Spearman’s rank correlations were computed between the functional gene diversity/abundances and environmental factors using the R package “Hmisc” (Frank, 2022). Non-metric multidimensional scaling (NMDS) was applied to visualize the difference in functional gene compositions among the studied samples based on Bray–Curtis dissimilarity; and the function of *envfit* in the “vegan” package was used to identify significant (*p* < 0.05) environmental factors that influence functional gene composition (Oksanen et al., 2020). Subsequently, the significant environmental factors were chosen for random forest model analysis (Breiman, 2001). The importance of the selected factors in random forest models was assessed by the extent to which the mean square error (MSE) increased. Lastly, the goodness and significance of the final random forest model was evaluated by using *rf.significance* function in “rfUtilities” package (Evans and Murphy, 2018).

## Results

### Environmental parameters of the studied samples

The basic environmental parameters of the studied samples were shown in the Supplementary Table S1. Briefly, the salinity and pH of the sediment pore water were 0.7–31.5 g L^−1^ and 7.9–9.7, respectively. The TOC, TN, and TP contents of the studied sediments were 1.7–45.2, 0.1–1.8, and 0.0–0.2 mg g^−1^, respectively. Salinity was negatively (*p* < 0.05) correlated with TOC and TN; TOC was positively correlated with TN and the ratio of carbon to nitrogen (Supplementary Figure S1).

### Diversity and compositions of the CAZy, nitrogen, and sulfur cycling genes

In this study, the total numbers of amino acid coding gene sequences predicted by Prodigal ranged 393,357-1,241,878 among the studied samples. The total number of the CAZy, nitrogen, and sulfur cycling genes ranged 36,465-132,866, 5,922-21,802, and 19,963-75,834, respectively; and these genes were, respectively, classified to 373 CAZy, 67 nitrogen, and 204 sulfur cycling gene families. The Shannon diversity indices of the CAZy, nitrogen, and sulfur cycling genes ranged 4.134–4.387, 2.980–3.240, and 3.980–4.085, respectively.

**Figure 1:**
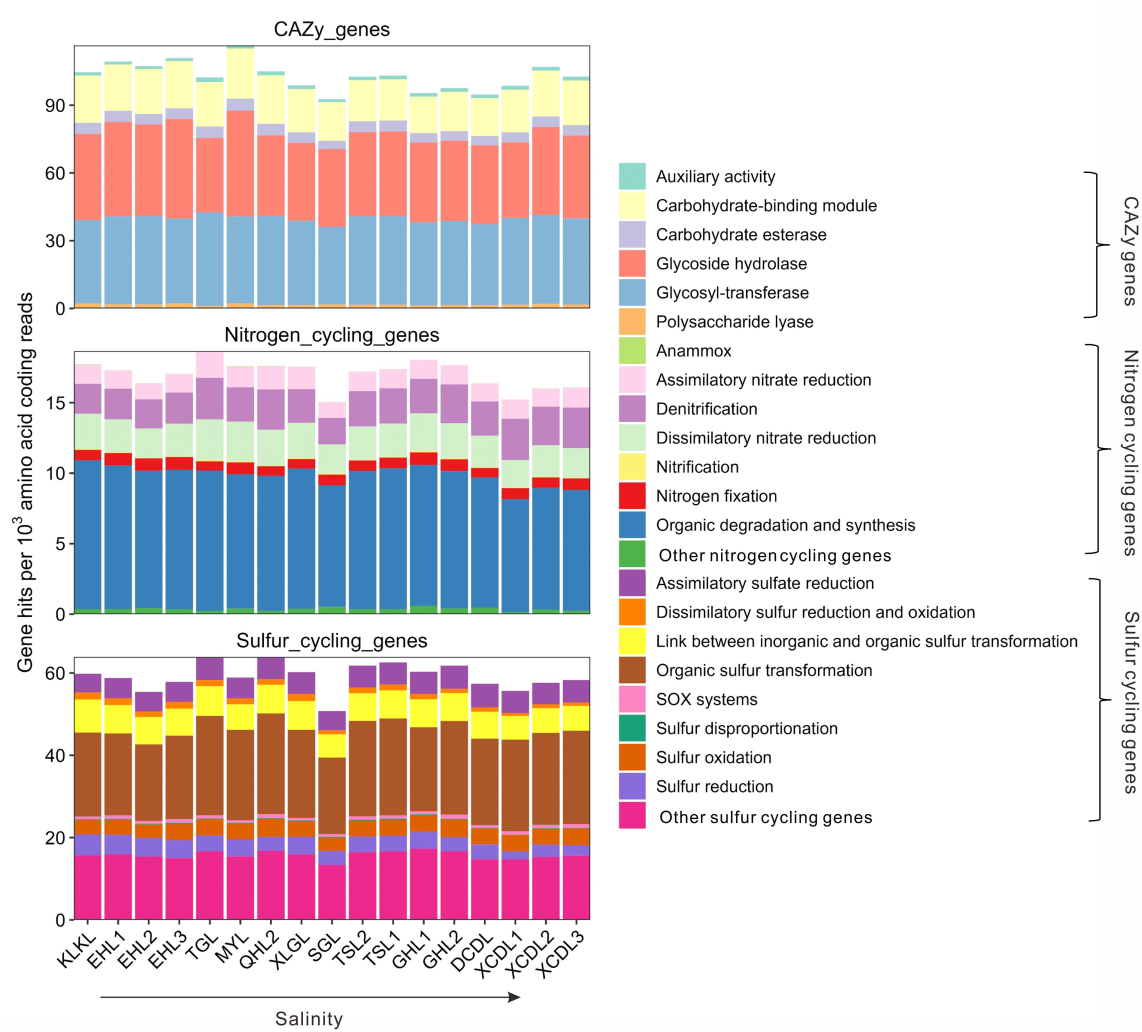
Relative abundances of the CAZy, nitrogen, and sulfur cycling gene categories in the studied lakes sediments.

The relative abundances of the CAZy genes in the studied samples ranged 92.7–116.5 per 1,000 amino acid coding gene reads (Figure 1). Among the CAZy genes, carbohydrate-binding module (CBM), glycoside hydrolase (GH), and glycosyl-transferase (GT) genes were dominant gene categories (>15 per 1,000 amino acid coding reads, Figure 1), and they accounted for 16.1–22.2, 31.6–45.5, and 35.5–42.7 per 1,000 amino acid coding gene reads, respectively (Figure 1). The relative abundance of auxiliary activity (AA), polysaccharide lyase (PL), and carbohydrate esterase (CE) genes was relatively low (<10 per 1,000 amino acid coding gene reads, Figure 1). The relative abundances of nitrogen cycling genes in the studied samples ranged 15.1–18.7 per 1,000 amino acid coding gene reads (Figure 1), with the gene category of organic degradation and synthesis having the highest relative abundance (ranging 8.0–10.6 per 1,000 amino acid coding reads, Figure 1). The relative abundances of sulfur cycling genes in the studied samples ranged 50.8–63.9 per 1,000 amino acid coding reads (Figure 1), with the gene category of organic sulfur transformation having the highest relative abundance (ranging 18.6–24.5 per 1,000 amino acid coding reads, Figure 1).

**Figure 2:**
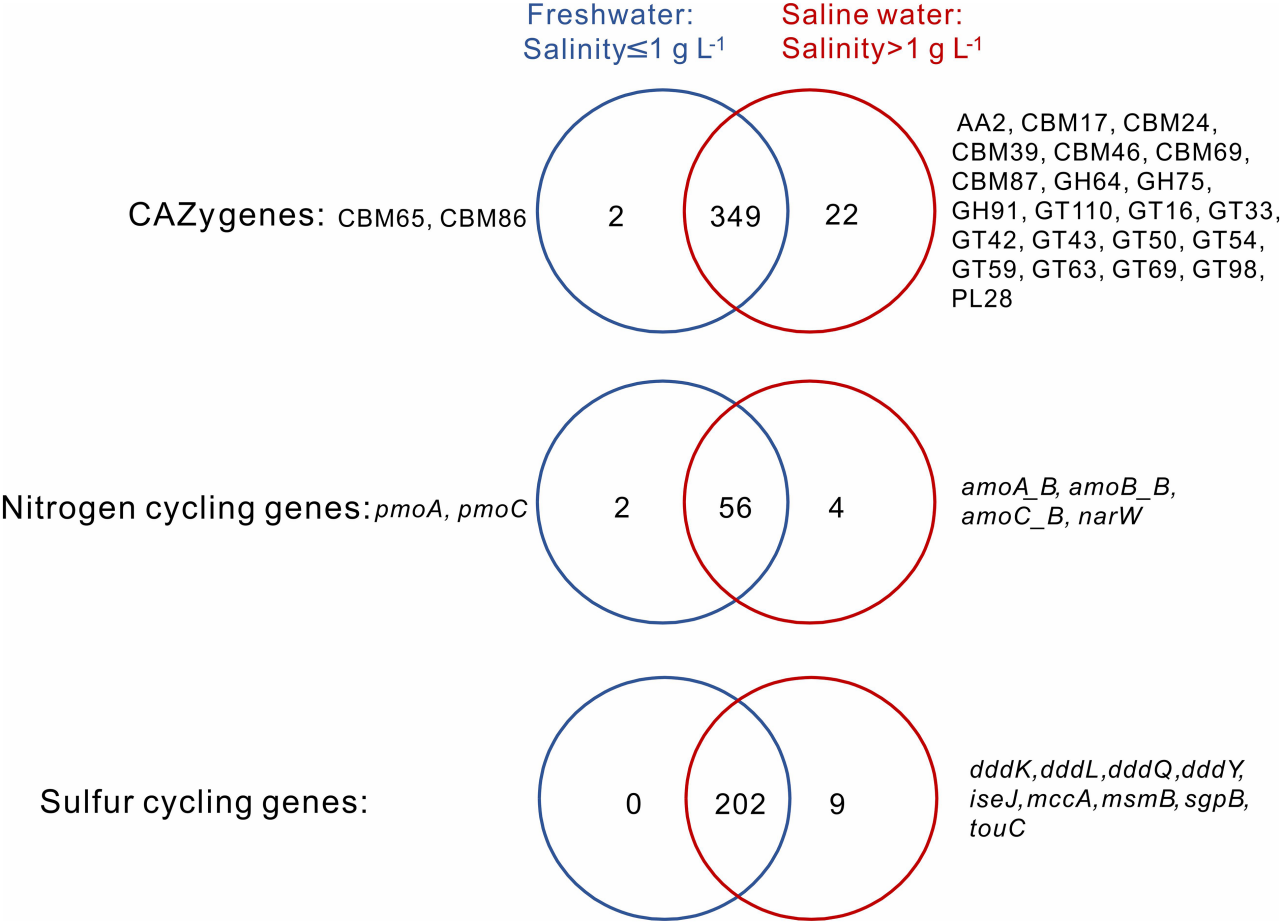
Venn diagrams show the numbers of unique and shared CAZy, nitrogen and sulfur cycling genes between the sediment samples from freshwater (salinity ≤ 1 g L^−1^) and saline (salinity >1 g L^−1^) lakes. Names of unique genes are labelled on both sides of the Venn diagrams.

A total of 349 CAZy, 56 nitrogen, and 202 sulfur cycling gene families were shared between the sediment samples from freshwater (salinity≤1 g L^−1^) and saline (salinity>1 g L^−1^) lakes, and they, respectively, accounted for 93.6, 90.3, and 95.7% of total numbers of the CAZy, nitrogen, and sulfur cycling gene families detected in this study (Figure 2). For the CAZy genes, totally 2 (i.e., CBM65, CBM86) and 22 unique genes (i.e., AA2, CBM17, CBM24, CBM39, CBM46, CBM69, CBM87, GH64, GH75, GH91, GT110, GT16, GT33, GT42, GT43, GT50, GT54, GT59, GT63, GT69, GT98, PL28) were present in the sediment samples from freshwater and saline lakes, respectively (Figure 2); for the nitrogen cycling genes, totally 2 unique genes (i.e., *pmoA*, *pmoC*) and 4 (i.e., *amoA_B*, *amoB_B*, *amoC_B*, *narW*) were present in the sediment samples from freshwater and saline lakes, respectively (Figure 2); for the sulfur cycling genes, there was none unique gene observed in the freshwater lake sediment samples, while 9 unique genes (i.e., *dddK*, *dddL*, *dddQ*, *dddY*, *iseJ*, *mccA*, *msmB*, *sgpB*, *touC*) were present in the saline lake samples (Figure 2).

### Taxonomic compositions of microbial communities that carry CAZy, nitrogen, and sulfur cycling genes

Microbial communities that carry the CAZy, nitrogen, and sulfur cycling genes were mainly affiliated with *Alphaproteobacteria*, *Bacteroidia*, *Bataproteobacteria*, *Clostridia*, *Cytophagia*, *Deltaproteobacteria*, *Flavobacteriia*, and *Gammaproteobacteria* (Supplementary Figure S2); and a large proportion (average relative abundance >20%) of these microbial communities were unclassified at the class level (Figure 2). Furthermore, the relative abundances of the microbial groups that carry the CAZy, nitrogen, and sulfur cycling genes were significantly correlated with environmental factors including salinity, TOC, TN, and TP; and the correlations of these microbial groups with salinity were higher than that with other measured factors (e.g., TOC, TN, and TP; Supplementary Figure S3). For example, for the microbial communities carrying the CAZy, nitrogen, and sulfur cycling genes, the relative abundances of the *Alphaproteobacteria*, *Balneolia*, and *Gammaproteobacteria* were all positively correlated with salinity; while negative correlations were observed between the abundances of salinity (Supplementary Figure S3).

### Spearman correlations between diversity/relative abundances of biogeochemical genes and environmental factors

**Figure 3:**
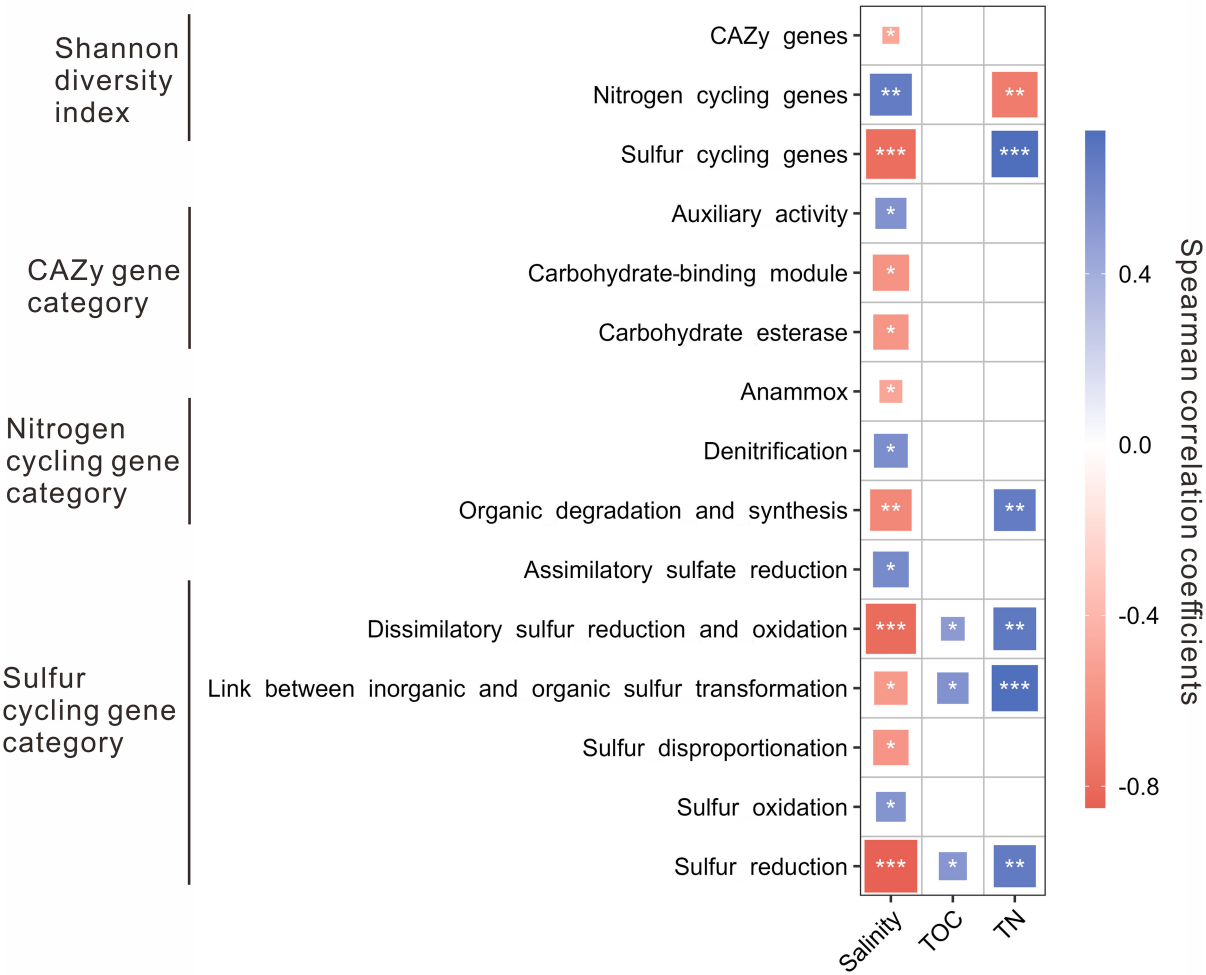
Spearman correlations between environmental factors (e.g., salinity, TOC, and TN) and the Shannon diversity of genes/relative abundances of gene categories related to the CAZy, nitrogen, and sulfur cycling in the studied lake sediments. Only significant (*p* < 0.05) correlations are shown in the figure. *p < 0.05, **p < 0.01, ***p < 0.001.

The Shannon diversity indices of nitrogen cycling genes were positively correlated with salinity, while that of the CAZy and sulfur cycling genes was negatively correlated with salinity. Besides, the Shannon diversity indices of sulfur cycling genes were positively correlated with TN content, while that of nitrogen cycling genes was negatively correlated with TN content (Figure 3). For the CAZy genes, the relative abundance of AA gene category was positively correlated with salinity, while that of CBM and CE gene categories were negatively correlated with salinity (Figure 3). For the nitrogen cycling genes, the relative abundance of denitrification gene category was positively correlated with salinity, while that of the gene categories involved in anammox and organic degradation and synthesis was negatively correlated with salinity (Figure 3); the relative abundance of the gene category related to organic degradation and synthesis was positively correlated with TN content. For sulfur cycling genes, positive correlations were present between salinity and the relative abundance of the gene categories involved in assimilatory sulfate reduction and sulfur oxidation, while negative correlations were present between salinity and the relative abundance of the gene categories related to dissimilatory sulfur reduction and oxidation, transformation between inorganic and organic sulfur, sulfur disproportionation, and sulfur reduction (Figure 3); positive correlations were shown between the contents of TOC and TN and the relative abundances of the gene categories related to dissimilatory sulfur reduction and oxidation, transformation between inorganic and organic sulfur, and sulfur reduction (Figure 3).

**Figure 4:**
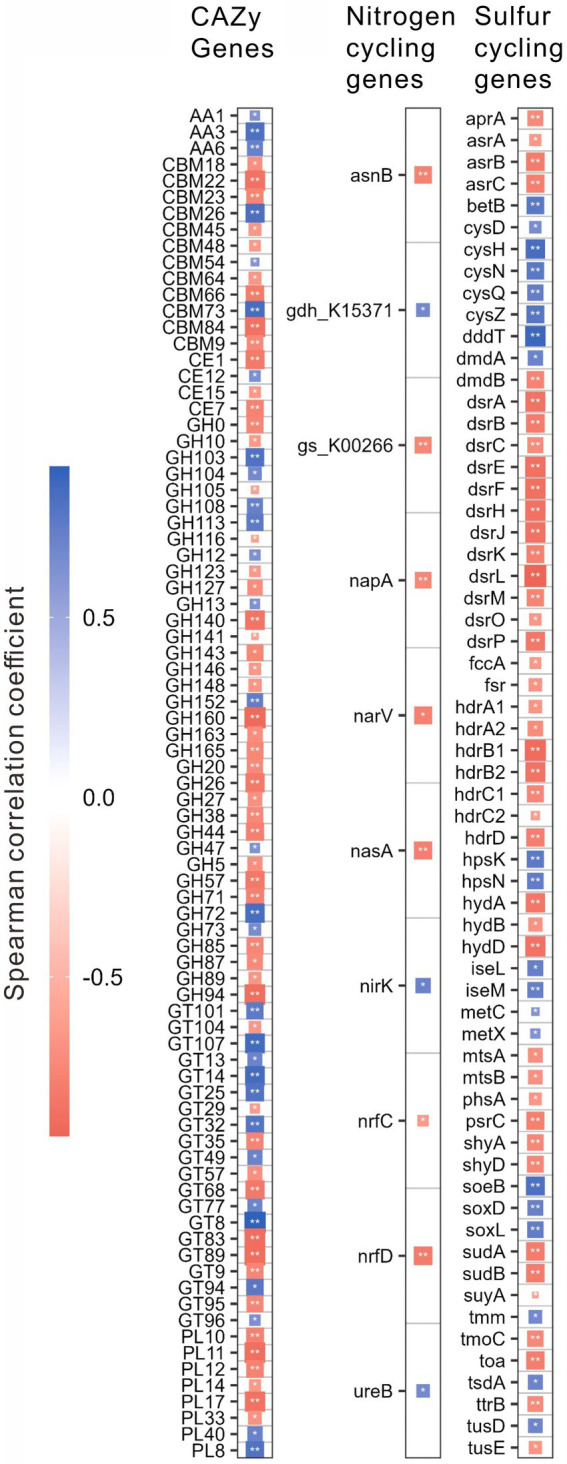
Spearman correlations between the relative abundances of the specifical genes related to the CAZy, nitrogen, and sulfur cycling and salinity in the studied lake sediments. Only significant (*p* < 0.01) correlations are shown in the figure. *p < 0.01, **p < 0.001.

For different functional genes, the relative abundances of 118 CAZy, 12 nitrogen, and 68 sulfur cycling genes were significantly (*p* < 0.01) correlated with salinity, pH, the contents of TOC, and TN, C/N or TP (Supplementary Table S2). Interestingly, the relative abundances of these genes were mostly (i.e., 83, 10, and 62 genes of CAZy, nitrogen, and sulfur cycling, respectively) correlated with salinity (Figure 4; Supplementary Table S2). Specifically, these CAZy genes included 3 AA genes, 4 CE genes, 12 CBM genes, 36 GH genes, 20 GT genes and 8 PL genes; the nitrogen cycling genes included one gene (i.e., *nasA*) of assimilatory nitrate reduction, 2 genes (i.e., *nrfC* and *nrfD*) of dissimilatory nitrate reduction, 3 genes (e.g., *narV*, *nirK*) of denitrification, 4 genes (e.g., *asnB*, *ureB*) of organic degradation and synthesis; the sulfur cycling genes contained 4 genes (e.g., *cysD*, *cysN*) of assimilatory sulfate reduction, 13 genes (e.g., *aprA*, *dsrL*) of dissimilatory sulfur reduction and oxidation, 11 genes (e.g., *hdrB*, *suyA*) of link between inorganic and organic sulfur transformation, 8 genes (e.g., *mtsA*, *betB*) of organic sulfur transformation, one gene (i.e., *soxD*) of SOX systems, one gene (i.e., *phsA*) of sulfur disproportionation, 3 genes (e.g., *fccA*, *soeB*) of sulfur oxidation, 3 genes (e.g., *hydA*, *shyA*) of sulfur reduction, and 8 unclassified sulfur cycling genes (Figure 4; Supplementary Table S2).

### Influences of environmental factors on the compositions of the CAZy, nitrogen, and sulfur cycling genes

**Figure 5:**
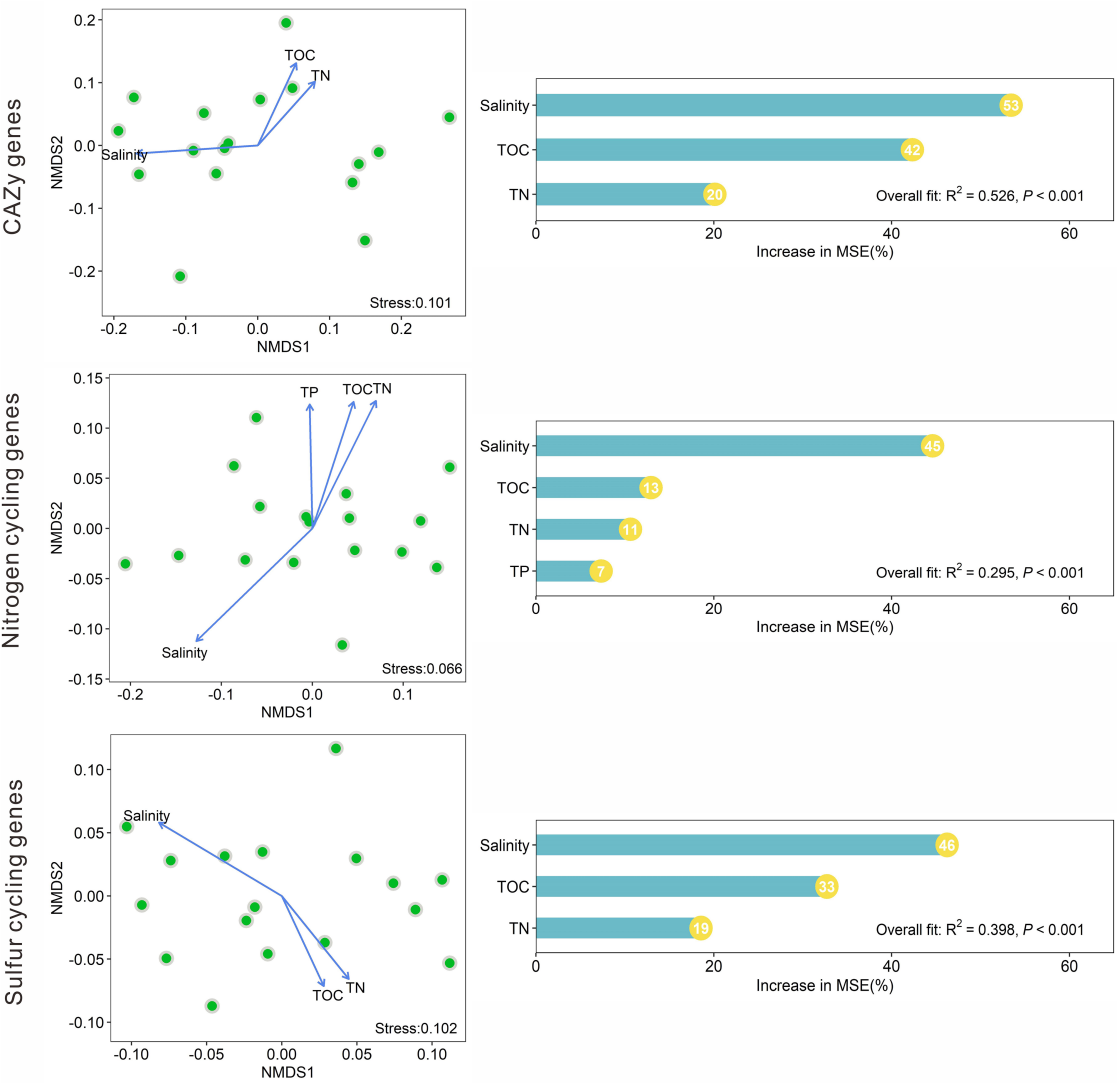
Nonmetric multidimensional scaling (NMDS) analyses of the CAZy, nitrogen, and sulfur cycling gene compositions based on Bray–Curtis dissimilarity among the studied lake sediments and their significant (*p* < 0.05) influencing factors, and the relative importance of these significant factors in influencing the compositions of the CAZy, nitrogen and sulfur cycling genes.

NMDS analysis indicated that the CAZy gene compositions in the studied lake sediments were significantly (*p* < 0.05) affected by salinity, TOC, and TN (Figure 5). The nitrogen cycling gene compositions were significantly affected by salinity, TOC, TN, and TP; and the sulfur cycling gene compositions were significantly influenced by salinity, TOC, and TN (Figure 5). Among the measured environmental factors, salinity was found to have the most important influence on the CAZy, nitrogen, and sulfur cycling gene compositions (Figure 5). The random forest models gave significant (*p* < 0.001) predictions of the CAZy, nitrogen, and sulfur cycling gene compositions with *R*^2^ values of 0.526, 0.295, and 0.398, respectively (Figure 5).

## Discussion

### Effect of salinity on the diversity of biogeochemical cycling genes

The negative correlations between salinity and the Shannon diversity indices of CAZy and sulfur cycling genes suggest that salinity may depress the diversity of CAZy and sulfur cycling genes in the present study. Such effect of salinity on the diversity of CAZy and sulfur cycling genes could be explained by the fact that microbial diversity is positively related to functional gene diversity in different ecosystems (Fierer et al., 2013; Bahram et al., 2018; Ruhl Ilona et al., 2022). Previous studies have indicated that microbial diversity decreases with increasing salinity in saline lake sediments (Jiang et al., 2006; Yang et al., 2016b; Liu et al., 2019). Thus with the increase of salinity, the diversity of CAZy and sulfur cycling genes possibly decreased synchronously with microbial diversity in the studied lake sediments. Moreover, the positive correlation between salinity and the Shannon diversity indices of nitrogen cycling genes suggest that salinity may enhance nitrogen cycling gene diversity in the studied lake sediments. This finding can be explained by the fact that many microorganisms cope with high salinity stress primarily by accumulating amino acid derivatives (e.g., glutamine, glutamate) in cells (Oren, 2008, 2011). Many functional genes (e.g., *glnA*, *asnB*, and *gs*_K00264) that participate in the synthesis of amino acid derivatives are classified as nitrogen cycling genes (Tu et al., 2019). Therefore, with increasing salinity, the presence of functional genes related to the synthesis of amino acid derivatives may be conductive to the enhancement of nitrogen cycling gene diversity in the studied lake sediments. In addition, TN may also significantly affect the diversity of nitrogen and sulfur cycling genes in the present study, as supported by significant correlations between TN content and the Shannon diversity indices of nitrogen and sulfur cycling genes. However, the underlying mechanisms of these TN effects remain unclear and await further investigation.

### Influence of environmental factors on the compositions of biogeochemical cycling genes

It is expected that environmental factors (e.g., salinity, TOC, TN, TP) significantly affect the compositions of the CAZy, nitrogen, and sulfur cycling genes in this study. These factors have long been recognized as significant influencing factors in the taxonomic composition of microbial communities in lakes. For instance, salinity can cause microbial physiological constraints on osmoregulation and energy (Oren, 2008; Rath et al., 2019), so it will select microbial communities and influence microbial distribution in lakes, resulting in distinct microbial community compositions in lakes with various salinity (Wu et al., 2006; Jiang et al., 2007; Wang et al., 2011; Logares et al., 2013; Liu et al., 2016; Yang et al., 2016b; Zhong et al., 2016; Ji et al., 2019). TOC acts as a substrate to support the growth of heterotrophic microbes that are dominant components of microbial community, thus it can influence microbial community composition in lakes (Hollister et al., 2010; Yang et al., 2016a). TN and TP are nutrient factors that affect microbial growth, so they can influence the composition of microbial communities in lakes (Yang et al., 2018; Liu et al., 2019; Sun et al., 2022). Previous studies have shown that microbial functional gene composition is closely related to the taxonomic composition of microbial community (Zimmerman et al., 2013; Wang et al., 2022). Indeed, the relative abundances of many microbial groups carrying the CAZy, nitrogen and sulfur cycling genes were significantly correlated with environmental factors such as salinity, TOC, TN, and TP (Supplementary Figure S3). Therefore, the significant influence of environmental factors on the compositions of CAZy, nitrogen, and sulfur cycling genes may result from the indirect effect of environmental factors on the taxonomic composition of microbial communities in the studied lake sediments.

It is notable that in the present study salinity was more important than other measured factors (e.g., TOC, TN, TP) in influencing the compositions of the CAZy, nitrogen, and sulfur cycling genes. This finding may be explained by the fact that salinity usually has a stronger influence on the microbial community composition in lake sediments than other environmental factors (Yang et al., 2016a; Zhong et al., 2016; Wang et al., 2021; Sun et al., 2022). There is a close relationship between microbial functional gene composition and taxonomic composition (Zimmerman et al., 2013; Wang et al., 2022). Therefore, it is not surprising that the distributions of microbial functional genes (e.g., CAZy, nitrogen, and sulfur cycling genes) were more susceptible to salinity than other measured factors in the studied samples. Mechanically, the survival of microorganisms under high salinity depends on their energy production. Many microorganisms (e.g., *Betaproteobacteria*) are limited to grow under high salinity due to energy constraints (Oren, 2011; Telesh et al., 2013; Vera-Gargallo and Ventosa, 2018). So it was reasonable to observe that *Betaproteobacteria*-associated functional genes (e.g., CAZy, nitrogen, and sulfur cycling genes) decreased with increasing salinity in this study. In addition, it was notable that the relative abundances of some functional gene categories (e.g., AA, denitrification, assimilatory sulfate reduction, sulfur oxidation) increased with increasing salinity. This finding may be explained by the fact microbial groups involved in AA, assimilatory sulfate reduction, denitrification, and sulfur oxidation were very active in high salinity environments (Oren, 2011; Sorokin et al., 2011; Yang et al., 2013b, 2021).

Interestingly, the relative abundance of many CAZy genes involved in the decomposition (e.g., AA3, AA6, CBM54, CBM73, CE12, GH12, GH103, GH104, PL8) of refractory organic matters (e.g., chitin, hemicellulose, peptidoglycan, lignin, xylan) increased with increasing salinity in the studied lake sediments, suggesting that microorganisms under high salinity may exploit refractory organic matter as energy sources for their growth. Indeed, it has been reported that at high salinity, many halotolerant/halophilic microbes possess multiple enzymes capable of degrading refractory organic matter derived from plant and microbial cell walls (Sorokin et al., 2015; Cortes-Tolalpa et al., 2018). One of our previous studies also displayed that the microbial community in high-salinity lakes had a stronger ability to use dissolved organic matter with high carbon number and high complexity than that in low salinity lakes (Yang et al., 2020b). Since the abundance of halotolerant/halophilic microorganisms usually increases with salinity, the abundance of their associated genes (e.g., the aforementioned CAZy genes) will also increase (Yang et al., 2021).

It is also remarkable that the relative abundance of functional genes involved in glutamate dehydrogenation (e.g., *gdh*_K15371), betaine synthesis (e.g., *dddT*, *betB*), and sulfur oxidation (e.g., *soeB*, *soxD, tsdA*) increased with increasing salinity in this study. Betaine and β-glutamate are typical organic osmotic solutes that aids microbial resistance to salinity, so microorganisms with synthesis function of such organic osmotic solutes may be highly active at high salinity (Oren, 2011). Besides, some reactions of sulfur oxidation are high energy-producing processes (Sorokin, 2008; Sorokin et al., 2011), so microorganisms involved in these sulfur oxidation processes may be more active at high salinity than at low salinity (Yang et al., 2013b). Therefore, it is reasonable to observe that the enrichment of functional genes associated with organic osmotic solute synthesis and sulfur oxidation with increasing salinity in the studied lake sediments.

In summary, salinity is an important factor shaping the diversity of the CAZy, nitrogen, and sulfur cycling genes in the studied lake sediments. The diversity of the CAZy and sulfur cycling genes decreased as salinity increased, whereas the diversity of the nitrogen cycling genes increased. The compositions of the CAZy, nitrogen, and sulfur cycling genes in the studied lake sediments were significantly influenced by environmental factors such as salinity, TOC, TN, and TP, with salinity having the greatest impact. Collectively, our study shed light on how salinity affects the diversity and composition of CAZy, nitrogen, and sulfur cycling genes in lacustrine ecosystems.

## Data availability statement

The datasets presented in this study can be found in online repositories. The names of the repository/repositories and accession number(s) can be found in the article/Supplementary material.

## Author contributions

HJ conceived this study. HJ, JY, WL, and BW collected the samples. BW performed the geochemical analysis. QL, ZH, and JY conducted the metagenomic analysis. QL, JY, and HJ drafted the manuscript. All authors reviewed the results and wrote, revised the manuscript.

## Funding

This research was supported by grants from the National Natural Science Foundation of China (Grant Nos. 92251304 and 41972317), the 111 Program (State Administration of Foreign Experts Affairs & the Ministry of Education of China, grant B18049), Introduction Project of High-Level Talents in Xinjiang Uygur Autonomous Region, the Second Tibetan Plateau Scientific Expedition and Research Program (STEP) (2019QZKK0805), the Science and Technology Plan Project of Qinghai Province (Grant No. 2022-ZJ-Y08), and State Key Laboratory of Biogeology and Environmental Geology, CUG (No. GBL11805).

## Conflict of interest

The authors declare that the research was conducted in the absence of any commercial or financial relationships that could be construed as a potential conflict of interest.

## Publisher’s note

All claims expressed in this article are solely those of the authors and do not necessarily represent those of their affiliated organizations, or those of the publisher, the editors and the reviewers. Any product that may be evaluated in this article, or claim that may be made by its manufacturer, is not guaranteed or endorsed by the publisher.
