# Supplementary material for: Influence of salinity on the diversity and composition of carbohydrate metabolism, nitrogen and sulfur cycling genes in lake surface sediments
Source: Front Microbiol. 2022 Nov 28;13:1019010. doi: 10.3389/fmicb.2022.1019010 (PMC9742235; doi:10.3389/fmicb.2022.1019010)

Table S1 Environmental parameters of the studied samples

| Lake name | Sample ID | | GPS location | Salinity  (g L^-1^) | pH | TOC  (mg g^-1^) | TN  (mg g^-1^) | TP  (mg g^-1^) |
| --- | --- | --- | --- | --- | --- | --- | --- | --- |
| Keluke Lake | | KLKL | N 37.277279°  E 96.860500° | 0.7 | 8.2 | 36.389 | 1.642 | 0.205 |
| Erhai Lake | | EHL1 | N 36.568333° E 100.738333° | 0.9 | 8.6 | 18.280 | 1.009 | 0.138 |
| Erhai Lake | | EHL2 | N 36.568333° E 100.738333° | 0.9 | 8.6 | 19.163 | 0.693 | 0.084 |
| Erhai Lake | | EHL3 | N 36.568333° E 100.738333° | 0.9 | 8.6 | 23.154 | 0.886 | 0.101 |
| Tiangeli Lake | | TGL | N 36.523884°  E 96.195342° | 1.0 | 8.4 | 45.161 | 0.993 | 0.244 |
| Mangya Lake | | MYL | N 37.826790°  E 91.734770° | 2.1 | 7.9 | 15.246 | 0.162 | 0.106 |
| Qinghai Lake | | QHL2 | N 36.6333333° E 100.115000° | 12.1 | 8.7 | 22.130 | 0.558 | 0.120 |
| Xiligou Lake | | XLGL | N 36.833974°  E 98.419254° | 13.1 | 7.9 | 37.717 | 1.844 | 0.233 |
| Sugan Lake | | SGL | N 38.898385°  E 93.904198° | 16.6 | 8.5 | 7.962 | 0.460 | 0.134 |
| Tuosu Lake | | TSL2 | N 37.197800°  E 96.868100° | 17.24 | 9.6 | 2.338 | 0.207 | 0.125 |
| Tuosu Lake | | TSL1 | N 37.197800°  E 96.868100° | 17.28 | 9.7 | 2.672 | 0.222 | 0.142 |
| Gahai Lake | | GHL1 | N 36.968333° E 100.598333° | 25.1 | 8.8 | 30.294 | 0.358 | 0.145 |
| Gahai Lake | | GHL2 | N 36.968333° E 100.598333° | 25.1 | 8.8 | 10.977 | 0.917 | 0.104 |
| Dachaidan Lake | | DCDL | N 37.788382°  E 95.241492° | 25.2 | 7.9 | 41.693 | 0.745 | 0.109 |
| Xiaochaidan Lake | | XCDL1 | N 37.480000° E 95.436666° | 31.0 | 8.0 | 3.893 | 0.128 | 0.145 |
| Xiaochaidan Lake | | XCDL2 | N 37.480000° E 95.436666° | 31.0 | 8.0 | 1.671 | 0.160 | 0.152 |
| Xiaochaidan Lake | | XCDL3 | N 37.480000° E 95.436666° | 31.5 | 8.0 | 3.223 | 0.114 | 0.127 |

Table S2 Spearman correlation analyses between the relative abundances of specific functional genes and environmental factors in this study.

|  | Genes | Factors | R | *P* | Category |
| --- | --- | --- | --- | --- | --- |
| CAZy genes | GH160 | Salinity | -0.91581 | 0.00000 | Glycoside hydrolase |
| CAZy genes | GT89 | Salinity | -0.89792 | 0.00000 | Glycosyl-transferase |
| CAZy genes | GT83 | Salinity | -0.88930 | 0.00000 | Glycosyl-transferase |
| CAZy genes | GH94 | Salinity | -0.88807 | 0.00000 | Glycoside hydrolase |
| CAZy genes | PL11 | Salinity | -0.88807 | 0.00000 | Polysaccharide lyase |
| CAZy genes | PL17 | Salinity | -0.87577 | 0.00000 | Polysaccharide lyase |
| CAZy genes | CBM84 | Salinity | -0.87208 | 0.00001 | Carbohydrate-binding module |
| CAZy genes | CBM22 | Salinity | -0.86962 | 0.00001 | Carbohydrate-binding module |
| CAZy genes | GH140 | Salinity | -0.85855 | 0.00001 | Glycoside hydrolase |
| CAZy genes | GH57 | Salinity | -0.83272 | 0.00003 | Glycoside hydrolase |
| CAZy genes | GT68 | Salinity | -0.83272 | 0.00003 | Glycosyl-transferase |
| CAZy genes | GH26 | Salinity | -0.83149 | 0.00004 | Glycoside hydrolase |
| CAZy genes | CE1 | Salinity | -0.82780 | 0.00004 | Carbohydrate esterase |
| CAZy genes | GT71 | TP | -0.82675 | 0.00318 | Glycosyl-transferase |
| CAZy genes | CBM66 | Salinity | -0.81427 | 0.00007 | Carbohydrate-binding module |
| CAZy genes | GH110 | TN | -0.80392 | 0.00010 | Glycoside hydrolase |
| CAZy genes | GT30 | TOC | -0.79902 | 0.00012 | Glycosyl-transferase |
| CAZy genes | GH44 | Salinity | -0.79558 | 0.00023 | Glycoside hydrolase |
| CAZy genes | GH71 | Salinity | -0.79213 | 0.00015 | Glycoside hydrolase |
| CAZy genes | PL10 | Salinity | -0.78844 | 0.00017 | Polysaccharide lyase |
| CAZy genes | CE7 | Salinity | -0.78229 | 0.00021 | Carbohydrate esterase |
| CAZy genes | PL12 | Salinity | -0.78229 | 0.00021 | Polysaccharide lyase |
| CAZy genes | GH110 | TOC | -0.77451 | 0.00026 | Glycoside hydrolase |
| CAZy genes | GH66 | pH | -0.77448 | 0.00115 | Glycoside hydrolase |
| CAZy genes | CBM23 | Salinity | -0.77368 | 0.00027 | Carbohydrate-binding module |
| CAZy genes | GT23 | TOC | -0.77206 | 0.00028 | Glycosyl-transferase |
| CAZy genes | GT35 | Salinity | -0.76876 | 0.00031 | Glycosyl-transferase |
| CAZy genes | GH0 | Salinity | -0.76384 | 0.00036 | Glycoside hydrolase |
| CAZy genes | GT9 | Salinity | -0.76261 | 0.00037 | Glycosyl-transferase |
| CAZy genes | GH85 | Salinity | -0.76138 | 0.00038 | Glycoside hydrolase |
| CAZy genes | GT95 | Salinity | -0.76015 | 0.00040 | Glycosyl-transferase |
| CAZy genes | CBM27 | TN | -0.75735 | 0.00043 | Carbohydrate-binding module |
| CAZy genes | GH165 | Salinity | -0.75523 | 0.00046 | Glycoside hydrolase |
| CAZy genes | GH143 | Salinity | -0.75516 | 0.00113 | Glycoside hydrolase |
| CAZy genes | CBM9 | Salinity | -0.75031 | 0.00052 | Carbohydrate-binding module |
| CAZy genes | GH38 | Salinity | -0.74908 | 0.00054 | Glycoside hydrolase |
| CAZy genes | GH167 | TN | -0.74510 | 0.00060 | Glycoside hydrolase |
| CAZy genes | GH142 | TOC | -0.74510 | 0.00060 | Glycoside hydrolase |
| CAZy genes | GH20 | Salinity | -0.74170 | 0.00065 | Glycoside hydrolase |
| CAZy genes | AA10 | TN | -0.74020 | 0.00068 | Auxiliary activity |
| CAZy genes | CE12 | TN | -0.74020 | 0.00068 | Carbohydrate esterase |
| CAZy genes | GH158 | TOC | -0.73529 | 0.00077 | Glycoside hydrolase |
| CAZy genes | GH87 | Salinity | -0.73265 | 0.00125 | Glycoside hydrolase |
| CAZy genes | GH145 | TN | -0.72549 | 0.00098 | Glycoside hydrolase |
| CAZy genes | GH136 | TOC | -0.72304 | 0.00104 | Glycoside hydrolase |
| CAZy genes | GH145 | TOC | -0.72304 | 0.00104 | Glycoside hydrolase |
| CAZy genes | GH163 | Salinity | -0.72079 | 0.00110 | Glycoside hydrolase |
| CAZy genes | GH127 | Salinity | -0.71956 | 0.00113 | Glycoside hydrolase |
| CAZy genes | AA10 | TOC | -0.71814 | 0.00117 | Auxiliary activity |
| CAZy genes | PL29 | TOC | -0.71814 | 0.00117 | Polysaccharide lyase |
| CAZy genes | GH5 | Salinity | -0.71464 | 0.00127 | Glycoside hydrolase |
| CAZy genes | GH79 | pH | -0.71366 | 0.00415 | Glycoside hydrolase |
| CAZy genes | GH152 | TN | -0.71078 | 0.00138 | Glycoside hydrolase |
| CAZy genes | GT25 | TN | -0.71078 | 0.00138 | Glycosyl-transferase |
| CAZy genes | GT76 | TN | -0.71078 | 0.00138 | Glycosyl-transferase |
| CAZy genes | GT11 | TOC | -0.71078 | 0.00138 | Glycosyl-transferase |
| CAZy genes | PL40 | TN | -0.70882 | 0.00211 | Polysaccharide lyase |
| CAZy genes | GT57 | Salinity | -0.70726 | 0.00150 | Glycosyl-transferase |
| CAZy genes | GH16 | TOC | -0.70588 | 0.00154 | Glycoside hydrolase |
| CAZy genes | CBM18 | Salinity | -0.70459 | 0.00231 | Carbohydrate-binding module |
| CAZy genes | PL40 | TOC | -0.70294 | 0.00239 | Polysaccharide lyase |
| CAZy genes | GH128 | TN | -0.69853 | 0.00181 | Glycoside hydrolase |
| CAZy genes | GH27 | Salinity | -0.69742 | 0.00186 | Glycoside hydrolase |
| CAZy genes | GH136 | TN | -0.69118 | 0.00212 | Glycoside hydrolase |
| CAZy genes | AA0 | TOC | -0.68873 | 0.00223 | Auxiliary activity |
| CAZy genes | PL33 | Salinity | -0.68266 | 0.00253 | Polysaccharide lyase |
| CAZy genes | GT8 | TN | -0.67892 | 0.00273 | Glycosyl-transferase |
| CAZy genes | GT76 | TOC | -0.67892 | 0.00273 | Glycosyl-transferase |
| CAZy genes | GT25 | TOC | -0.67647 | 0.00287 | Glycosyl-transferase |
| CAZy genes | GH148 | Salinity | -0.67159 | 0.00315 | Glycoside hydrolase |
| CAZy genes | CBM26 | TN | -0.67157 | 0.00316 | Carbohydrate-binding module |
| CAZy genes | GH114 | TOC | -0.67157 | 0.00316 | Glycoside hydrolase |
| CAZy genes | AA0 | TN | -0.66912 | 0.00331 | Auxiliary activity |
| CAZy genes | CBM45 | Salinity | -0.66667 | 0.00347 | Carbohydrate-binding module |
| CAZy genes | GH89 | Salinity | -0.66667 | 0.00347 | Glycoside hydrolase |
| CAZy genes | CBM64 | Salinity | -0.66667 | 0.00664 | Carbohydrate-binding module |
| CAZy genes | GH142 | TN | -0.66176 | 0.00381 | Glycoside hydrolase |
| CAZy genes | PL8 | TOC | -0.65686 | 0.00417 | Polysaccharide lyase |
| CAZy genes | GT104 | Salinity | -0.65683 | 0.00418 | Glycosyl-transferase |
| CAZy genes | GH146 | Salinity | -0.65437 | 0.00437 | Glycoside hydrolase |
| CAZy genes | GH158 | TN | -0.65196 | 0.00457 | Glycoside hydrolase |
| CAZy genes | PL14 | Salinity | -0.64945 | 0.00478 | Polysaccharide lyase |
| CAZy genes | CE15 | Salinity | -0.64822 | 0.00489 | Carbohydrate esterase |
| CAZy genes | GT101 | TOC | -0.64706 | 0.00674 | Glycosyl-transferase |
| CAZy genes | CBM48 | Salinity | -0.64576 | 0.00511 | Carbohydrate-binding module |
| CAZy genes | GH10 | Salinity | -0.64576 | 0.00511 | Glycoside hydrolase |
| CAZy genes | GH16 | TN | -0.64461 | 0.00521 | Glycoside hydrolase |
| CAZy genes | GH123 | Salinity | -0.64453 | 0.00522 | Glycoside hydrolase |
| CAZy genes | GH103 | TN | -0.64216 | 0.00545 | Glycoside hydrolase |
| CAZy genes | GT101 | TN | -0.63824 | 0.00780 | Glycosyl-transferase |
| CAZy genes | GH114 | TN | -0.63480 | 0.00619 | Glycoside hydrolase |
| CAZy genes | GH128 | TOC | -0.63480 | 0.00619 | Glycoside hydrolase |
| CAZy genes | GT23 | TN | -0.63235 | 0.00645 | Glycosyl-transferase |
| CAZy genes | CE12 | TOC | -0.62990 | 0.00673 | Carbohydrate esterase |
| CAZy genes | GH149 | TOC | -0.62990 | 0.00673 | Glycoside hydrolase |
| CAZy genes | GT29 | Salinity | -0.62608 | 0.00717 | Glycosyl-transferase |
| CAZy genes | GT30 | TN | -0.62255 | 0.00761 | Glycosyl-transferase |
| CAZy genes | PL8 | TN | -0.62255 | 0.00761 | Polysaccharide lyase |
| CAZy genes | GH113 | TOC | -0.62010 | 0.00792 | Glycoside hydrolase |
| CAZy genes | GH43 | TP | -0.61312 | 0.00887 | Glycoside hydrolase |
| CAZy genes | GH105 | Salinity | -0.61132 | 0.00912 | Glycoside hydrolase |
| CAZy genes | GH116 | Salinity | -0.60886 | 0.00949 | Glycoside hydrolase |
| CAZy genes | GH147 | TOC | -0.60784 | 0.00964 | Glycoside hydrolase |
| CAZy genes | GT11 | TN | -0.60784 | 0.00964 | Glycosyl-transferase |
| CAZy genes | GH141 | Salinity | -0.60763 | 0.00967 | Glycoside hydrolase |
| CAZy genes | GT10 | TP | -0.60699 | 0.00977 | Glycosyl-transferase |
| CAZy genes | GT87 | pH | 0.60614 | 0.00990 | Glycoside hydrolase |
| CAZy genes | GH46 | TN | 0.60784 | 0.00964 | Glycoside hydrolase |
| CAZy genes | GH57 | TN | 0.61029 | 0.00927 | Glycoside hydrolase |
| CAZy genes | GT24 | TN | 0.61275 | 0.00892 | Glycosyl-transferase |
| CAZy genes | CBM54 | Salinity | 0.61378 | 0.00877 | Carbohydrate-binding module |
| CAZy genes | GT22 | pH | 0.61841 | 0.00814 | Glycoside hydrolase |
| CAZy genes | AA1 | Salinity | 0.62731 | 0.00703 | Auxiliary activity |
| CAZy genes | GH47 | Salinity | 0.62731 | 0.00703 | Glycoside hydrolase |
| CAZy genes | GH13 | Salinity | 0.62854 | 0.00688 | Glycoside hydrolase |
| CAZy genes | GT81 | TN | 0.63235 | 0.00645 | Glycosyl-transferase |
| CAZy genes | GT64 | TN | 0.63725 | 0.00593 | Glycosyl-transferase |
| CAZy genes | GH12 | Salinity | 0.63838 | 0.00582 | Glycoside hydrolase |
| CAZy genes | GT96 | Salinity | 0.63961 | 0.00569 | Glycosyl-transferase |
| CAZy genes | CE12 | Salinity | 0.64453 | 0.00522 | Carbohydrate esterase |
| CAZy genes | GH94 | TN | 0.64951 | 0.00478 | Glycoside hydrolase |
| CAZy genes | GH73 | Salinity | 0.66175 | 0.00381 | Glycoside hydrolase |
| CAZy genes | GH104 | Salinity | 0.67774 | 0.00279 | Glycoside hydrolase |
| CAZy genes | GH107 | TP | 0.67844 | 0.00386 | Glycoside hydrolase |
| CAZy genes | GT68 | TN | 0.68873 | 0.00223 | Glycosyl-transferase |
| CAZy genes | GT95 | TOC | 0.69363 | 0.00201 | Glycosyl-transferase |
| CAZy genes | GT74 | TOC | 0.69670 | 0.00563 | Glycosyl-transferase |
| CAZy genes | GT95 | TN | 0.70098 | 0.00172 | Glycosyl-transferase |
| CAZy genes | GT77 | Salinity | 0.70726 | 0.00150 | Glycosyl-transferase |
| CAZy genes | GT13 | Salinity | 0.70972 | 0.00142 | Glycosyl-transferase |
| CAZy genes | GT49 | Salinity | 0.71341 | 0.00130 | Glycosyl-transferase |
| CAZy genes | PL40 | Salinity | 0.71776 | 0.00174 | Polysaccharide lyase |
| CAZy genes | GH76 | TN | 0.72059 | 0.00110 | Glycoside hydrolase |
| CAZy genes | GH76 | TOC | 0.72549 | 0.00098 | Glycoside hydrolase |
| CAZy genes | AA6 | Salinity | 0.72940 | 0.00089 | Auxiliary activity |
| CAZy genes | GH108 | Salinity | 0.73186 | 0.00084 | Glycoside hydrolase |
| CAZy genes | GH152 | Salinity | 0.74539 | 0.00059 | Glycoside hydrolase |
| CAZy genes | GH113 | Salinity | 0.76261 | 0.00037 | Glycoside hydrolase |
| CAZy genes | GT101 | Salinity | 0.76640 | 0.00053 | Glycosyl-transferase |
| CAZy genes | GT94 | Salinity | 0.77895 | 0.00283 | Glycosyl-transferase |
| CAZy genes | GT74 | TN | 0.79780 | 0.00063 | Glycosyl-transferase |
| CAZy genes | GT32 | Salinity | 0.80074 | 0.00011 | Glycosyl-transferase |
| CAZy genes | GT25 | Salinity | 0.80935 | 0.00008 | Glycosyl-transferase |
| CAZy genes | PL8 | Salinity | 0.80935 | 0.00008 | Polysaccharide lyase |
| CAZy genes | GH103 | Salinity | 0.81550 | 0.00007 | Glycoside hydrolase |
| CAZy genes | AA3 | Salinity | 0.82411 | 0.00005 | Auxiliary activity |
| CAZy genes | CBM26 | Salinity | 0.82903 | 0.00004 | Carbohydrate-binding module |
| CAZy genes | GH72 | Salinity | 0.83887 | 0.00003 | Glycoside hydrolase |
| CAZy genes | CBM73 | Salinity | 0.84625 | 0.00002 | Carbohydrate-binding module |
| CAZy genes | GT14 | Salinity | 0.85609 | 0.00001 | Glycosyl-transferase |
| CAZy genes | GT107 | Salinity | 0.85978 | 0.00001 | Glycosyl-transferase |
| CAZy genes | GT8 | Salinity | 0.91760 | 0.00000 | Glycosyl-transferase |
| Nitrogen cycling genes | *nrfD* | Salinity | -0.81919 | 0.00006 | Dissimilatory nitrate reduction |
| Nitrogen cycling genes | *nasA* | Salinity | -0.80074 | 0.00011 | Assimilatory nitrate reduction |
| Nitrogen cycling genes | *narV* | Salinity | -0.79007 | 0.00131 | Denitrification |
| Nitrogen cycling genes | *asnB* | Salinity | -0.78229 | 0.00021 | Organic degradation and synthesis |
| Nitrogen cycling genes | gs_K00266 | Salinity | -0.76138 | 0.00038 | Organic degradation and synthesis |
| Nitrogen cycling genes | *napA* | Salinity | -0.75277 | 0.00049 | Denitrification |
| Nitrogen cycling genes | *nrfB* | TOC | -0.74176 | 0.00370 | Dissimilatory nitrate reduction |
| Nitrogen cycling genes | gs_K00265 | TN | -0.64706 | 0.00499 | Organic degradation and synthesis |
| Nitrogen cycling genes | *nrfC* | Salinity | -0.63961 | 0.00569 | Dissimilatory nitrate reduction |
| Nitrogen cycling genes | *nifH* | TOC | -0.61275 | 0.00892 | Nitrogen fixation |
| Nitrogen cycling genes | *napA* | TN | 0.61520 | 0.00858 | Denitrification |
| Nitrogen cycling genes | gs_K00266 | TN | 0.62010 | 0.00792 | Organic degradation and synthesis |
| Nitrogen cycling genes | *nasA* | TN | 0.65686 | 0.00417 | Assimilatory nitrate reduction |
| Nitrogen cycling genes | *ureB* | Salinity | 0.67405 | 0.00301 | Organic degradation and synthesis |
| Nitrogen cycling genes | gdh_K15371 | Salinity | 0.68389 | 0.00247 | Organic degradation and synthesis |
| Nitrogen cycling genes | *nirK* | Salinity | 0.71833 | 0.00116 | Denitrification |
| Sulfur cycling genes | *dsrL* | Salinity | -0.94097 | 0.00000 | Dissimilatory sulfur reduction and oxidation |
| Sulfur cycling genes | *hdrB1* | Salinity | -0.90776 | 0.00000 | Linkages between inorganic and organic sulfur transformation |
| Sulfur cycling genes | *dsrE* | Salinity | -0.88069 | 0.00000 | Dissimilatory sulfur reduction and oxidation |
| Sulfur cycling genes | *dsrF* | Salinity | -0.87823 | 0.00000 | Dissimilatory sulfur reduction and oxidation |
| Sulfur cycling genes | *dsrH* | Salinity | -0.87577 | 0.00000 | Dissimilatory sulfur reduction and oxidation |
| Sulfur cycling genes | *dsrJ* | Salinity | -0.87298 | 0.00001 | Dissimilatory sulfur reduction and oxidation |
| Sulfur cycling genes | *hydD* | Salinity | -0.87208 | 0.00001 | Sulfur reduction |
| Sulfur cycling genes | *dsrA* | Salinity | -0.86347 | 0.00001 | Dissimilatory sulfur reduction and oxidation |
| Sulfur cycling genes | *hdrB2* | Salinity | -0.86347 | 0.00001 | Linkages between inorganic and organic sulfur transformation |
| Sulfur cycling genes | *hydA* | Salinity | -0.84010 | 0.00002 | Sulfur reduction |
| Sulfur cycling genes | *dsrP* | Salinity | -0.83641 | 0.00003 | Dissimilatory sulfur reduction and oxidation |
| Sulfur cycling genes | *dsrB* | Salinity | -0.81673 | 0.00006 | Dissimilatory sulfur reduction and oxidation |
| Sulfur cycling genes | *asrB* | Salinity | -0.81427 | 0.00007 | Sulfur reduction |
| Sulfur cycling genes | *sudA* | Salinity | -0.81058 | 0.00008 | Sulfur reduction |
| Sulfur cycling genes | *sudB* | Salinity | -0.81058 | 0.00008 | Sulfur reduction |
| Sulfur cycling genes | *toa* | Salinity | -0.80689 | 0.00009 | Organic sulfur transformation |
| Sulfur cycling genes | *hdrD* | Salinity | -0.80443 | 0.00010 | Linkages between inorganic and organic sulfur transformation |
| Sulfur cycling genes | *asrC* | Salinity | -0.79951 | 0.00012 | Sulfur reduction |
| Sulfur cycling genes | *psrC* | Salinity | -0.79582 | 0.00013 | Sulfur reduction |
| Sulfur cycling genes | *dsrK* | Salinity | -0.79090 | 0.00016 | Dissimilatory sulfur reduction and oxidation |
| Sulfur cycling genes | *dmdB* | Salinity | -0.77860 | 0.00023 | Organic sulfur transformation |
| Sulfur cycling genes | *dsrM* | Salinity | -0.77368 | 0.00027 | Dissimilatory sulfur reduction and oxidation |
| Sulfur cycling genes | *hdrC1* | Salinity | -0.76261 | 0.00037 | Linkages between inorganic and organic sulfur transformation |
| Sulfur cycling genes | *shyD* | Salinity | -0.75769 | 0.00043 | Sulfur reduction |
| Sulfur cycling genes | *shyA* | Salinity | -0.75154 | 0.00050 | Sulfur reduction |
| Sulfur cycling genes | *tmoC* | Salinity | -0.74785 | 0.00056 | Linkages between inorganic and organic sulfur transformation |
| Sulfur cycling genes | *tauZ* | TOC | -0.73775 | 0.00072 | Unclassified |
| Sulfur cycling genes | *dsrC* | Salinity | -0.73678 | 0.00074 | Dissimilatory sulfur reduction and oxidation |
| Sulfur cycling genes | *aprA* | Salinity | -0.73309 | 0.00081 | Dissimilatory sulfur reduction and oxidation |
| Sulfur cycling genes | *ttrB* | Salinity | -0.72940 | 0.00089 | Sulfur reduction |
| Sulfur cycling genes | *tmoF* | C/N | -0.72353 | 0.00153 | Linkages between inorganic and organic sulfur transformation |
| Sulfur cycling genes | *hdrA2* | Salinity | -0.72325 | 0.00103 | Linkages between inorganic and organic sulfur transformation |
| Sulfur cycling genes | *doxD* | TN | -0.71329 | 0.00920 | Sulfur oxidation |
| Sulfur cycling genes | *hpsK* | TN | -0.71078 | 0.00138 | Unclassified |
| Sulfur cycling genes | *mtsA* | Salinity | -0.70726 | 0.00150 | Organic sulfur transformation |
| Sulfur cycling genes | *tauZ* | TN | -0.70098 | 0.00172 | Unclassified |
| Sulfur cycling genes | *mtsB* | Salinity | -0.69865 | 0.00181 | Organic sulfur transformation |
| Sulfur cycling genes | *hydB* | Salinity | -0.69127 | 0.00212 | Sulfur reduction |
| Sulfur cycling genes | *hdrA1* | Salinity | -0.67774 | 0.00279 | Linkages between inorganic and organic sulfur transformation |
| Sulfur cycling genes | *fsr* | Salinity | -0.67774 | 0.00279 | Sulfur reduction |
| Sulfur cycling genes | *cysD* | TN | -0.67402 | 0.00301 | Assimilatory sulfate reduction |
| Sulfur cycling genes | *tusE* | Salinity | -0.67036 | 0.00323 | Unclassified |
| Sulfur cycling genes | *phsA* | Salinity | -0.66544 | 0.00355 | Sulfur disproportionation |
| Sulfur cycling genes | *dsrO* | Salinity | -0.65560 | 0.00427 | Dissimilatory sulfur reduction and oxidation |
| Sulfur cycling genes | *asrA* | Salinity | -0.65191 | 0.00457 | Sulfur reduction |
| Sulfur cycling genes | *fccA* | Salinity | -0.64576 | 0.00511 | Sulfur oxidation |
| Sulfur cycling genes | *cysD* | TOC | -0.63235 | 0.00645 | Assimilatory sulfate reduction |
| Sulfur cycling genes | *hdrC2* | Salinity | -0.61747 | 0.00827 | Linkages between inorganic and organic sulfur transformation |
| Sulfur cycling genes | *metX* | TN | -0.61520 | 0.00858 | Linkages between inorganic and organic sulfur transformation |
| Sulfur cycling genes | *betC* | TOC | -0.61275 | 0.00892 | Organic sulfur transformation |
| Sulfur cycling genes | *suyA* | Salinity | -0.60640 | 0.00986 | Linkages between inorganic and organic sulfur transformation |
| Sulfur cycling genes | *hydB* | TN | 0.60784 | 0.00964 | Sulfur reduction |
| Sulfur cycling genes | *metC* | Salinity | 0.61132 | 0.00912 | Linkages between inorganic and organic sulfur transformation |
| Sulfur cycling genes | *rdsr* | pH | 0.61472 | 0.00864 | Dissimilatory sulfur reduction and oxidation |
| Sulfur cycling genes | *asrC* | TN | 0.61520 | 0.00858 | Sulfur reduction |
| Sulfur cycling genes | *dsrA* | TN | 0.62500 | 0.00730 | Dissimilatory sulfur reduction and oxidation |
| Sulfur cycling genes | *dsrL* | TN | 0.62500 | 0.00730 | Dissimilatory sulfur reduction and oxidation |
| Sulfur cycling genes | *hdrB2* | TN | 0.62500 | 0.00730 | Linkages between inorganic and organic sulfur transformation |
| Sulfur cycling genes | *metX* | Salinity | 0.62608 | 0.00717 | Linkages between inorganic and organic sulfur transformation |
| Sulfur cycling genes | *dsrF* | TN | 0.62745 | 0.00701 | Dissimilatory sulfur reduction and oxidation |
| Sulfur cycling genes | *sudB* | TN | 0.62745 | 0.00701 | Sulfur reduction |
| Sulfur cycling genes | *dsrH* | TN | 0.63235 | 0.00645 | Dissimilatory sulfur reduction and oxidation |
| Sulfur cycling genes | *dsrK* | TN | 0.63480 | 0.00619 | Dissimilatory sulfur reduction and oxidation |
| Sulfur cycling genes | *dsrO* | TN | 0.63480 | 0.00619 | Dissimilatory sulfur reduction and oxidation |
| Sulfur cycling genes | *hdrA1* | TN | 0.63480 | 0.00619 | Linkages between inorganic and organic sulfur transformation |
| Sulfur cycling genes | *ttrA* | C/N | 0.63480 | 0.00619 | Sulfur reduction |
| Sulfur cycling genes | *hdrA2* | TN | 0.64461 | 0.00521 | Linkages between inorganic and organic sulfur transformation |
| Sulfur cycling genes | *shyA* | TN | 0.64706 | 0.00499 | Sulfur reduction |
| Sulfur cycling genes | *asrA* | TN | 0.65196 | 0.00457 | Sulfur reduction |
| Sulfur cycling genes | *hydA* | TN | 0.65196 | 0.00457 | Sulfur reduction |
| Sulfur cycling genes | *cysD* | Salinity | 0.65560 | 0.00427 | Assimilatory sulfate reduction |
| Sulfur cycling genes | *tmm* | Salinity | 0.68020 | 0.00266 | Organic sulfur transformation |
| Sulfur cycling genes | *asrB* | TN | 0.68873 | 0.00223 | Sulfur reduction |
| Sulfur cycling genes | *dsrC* | TN | 0.69118 | 0.00212 | Dissimilatory sulfur reduction and oxidation |
| Sulfur cycling genes | *hydD* | TN | 0.69118 | 0.00212 | Sulfur reduction |
| Sulfur cycling genes | *dsrM* | TN | 0.69363 | 0.00201 | Dissimilatory sulfur reduction and oxidation |
| Sulfur cycling genes | *tusD* | Salinity | 0.70009 | 0.00366 | Unclassified |
| Sulfur cycling genes | *sat* | TN | 0.70343 | 0.00163 | Dissimilatory sulfur reduction and oxidation |
| Sulfur cycling genes | *tsdA* | Salinity | 0.71095 | 0.00138 | Sulfur oxidation |
| Sulfur cycling genes | *ssuB* | pH | 0.71288 | 0.00132 | Linkages between inorganic and organic sulfur transformation |
| Sulfur cycling genes | *dmdA* | Salinity | 0.71710 | 0.00120 | Organic sulfur transformation |
| Sulfur cycling genes | *soxD* | Salinity | 0.73063 | 0.00086 | SOX systems |
| Sulfur cycling genes | *iseL* | Salinity | 0.73265 | 0.00125 | Unclassified |
| Sulfur cycling genes | *iseM* | Salinity | 0.73555 | 0.00077 | Unclassified |
| Sulfur cycling genes | *cysQ* | Salinity | 0.74293 | 0.00063 | Assimilatory sulfate reduction |
| Sulfur cycling genes | *soxL* | Salinity | 0.74662 | 0.00058 | Unclassified |
| Sulfur cycling genes | *hpsN* | Salinity | 0.75031 | 0.00052 | Unclassified |
| Sulfur cycling genes | *sat* | TOC | 0.76716 | 0.00033 | Dissimilatory sulfur reduction and oxidation |
| Sulfur cycling genes | *hpsK* | Salinity | 0.76999 | 0.00030 | Unclassified |
| Sulfur cycling genes | *cysN* | Salinity | 0.77860 | 0.00023 | Assimilatory sulfate reduction |
| Sulfur cycling genes | *betB* | Salinity | 0.78352 | 0.00020 | Organic sulfur transformation |
| Sulfur cycling genes | *cysZ* | Salinity | 0.79213 | 0.00015 | Unclassfied |
| Sulfur cycling genes | *soeB* | Salinity | 0.82411 | 0.00005 | Sulfur oxidation |
| Sulfur cycling genes | *cysH* | Salinity | 0.84010 | 0.00002 | Assimilatory sulfate reduction |
| Sulfur cycling genes | *dddT* | Salinity | 0.88069 | 0.00000 | Organic sulfur transformation |

Fig. S1 Spearman correlations between environmental factors

Fig. S2 Taxonomic compositions of microbial communities that carry the CAZy, nitrogen, and sulfur cycling genes in the studied sediment samples.


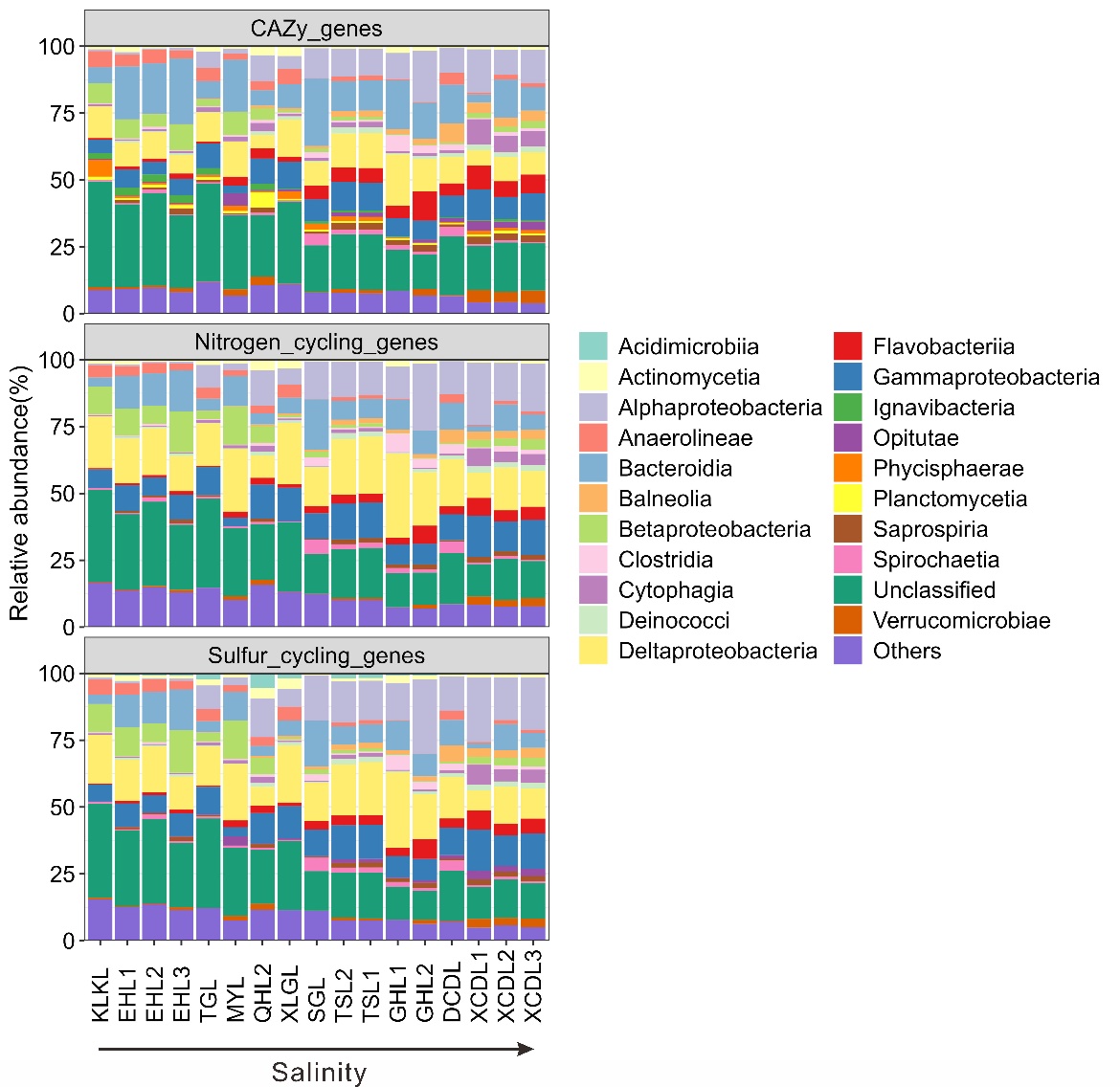


Fig. S3 Spearman correlations between environmental factors (e.g., salinity, TOC, TN) and the relative abundances of dominant microbial communities (average relative abundance > 1%) that carry the CAZy, nitrogen, and sulfur cycling in the studied lake sediments. Only significant (*P* <0.05) correlations are shown in the figure. *: *P* < 0.05, **: *P* <0.01, ***: *P*<0.001


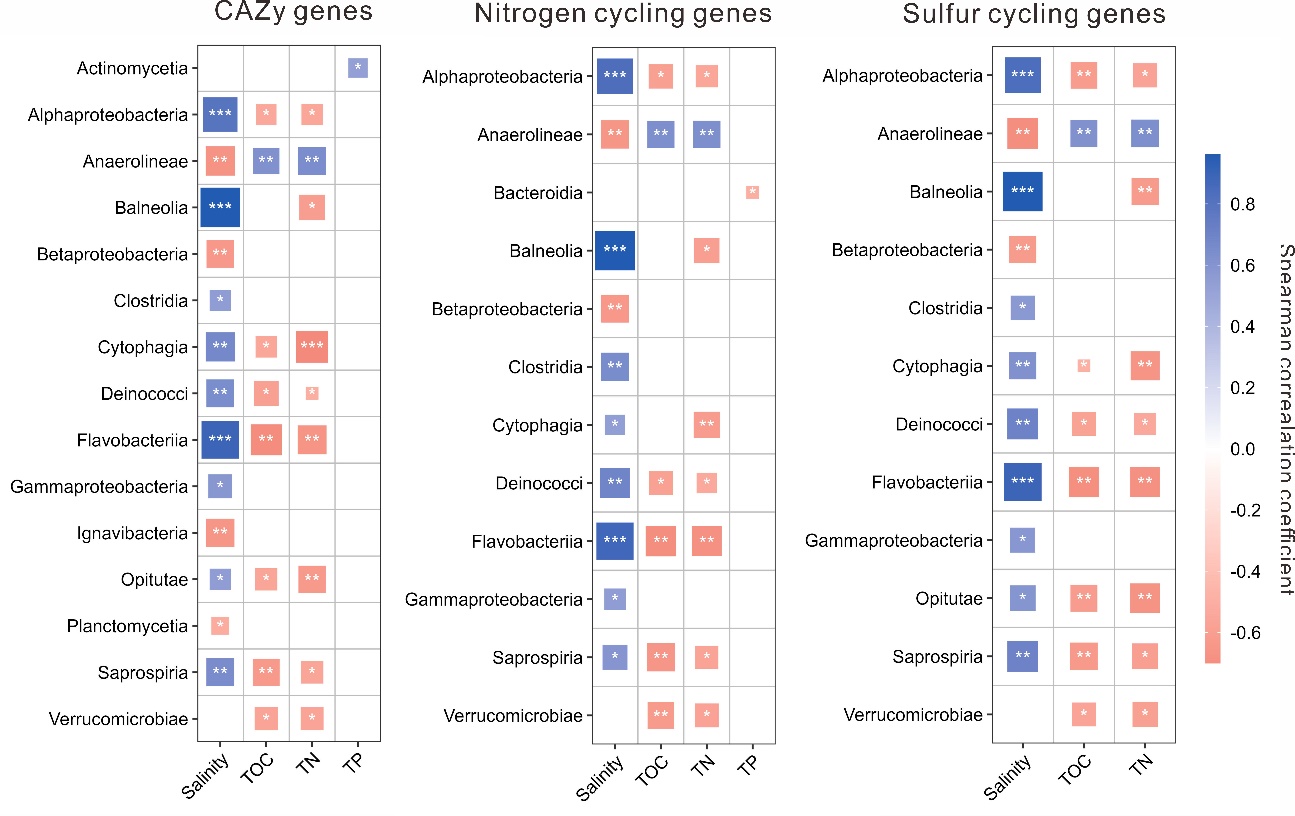

Supplement: Supplementary file 1 [file Data_Sheet_1.docx]
